# Long-Term Observation of SARS-CoV-2 Vaccination Response upon High Efficacy Treatment in Multiple Sclerosis—A Real-World Scenario

**DOI:** 10.3390/vaccines12030296

**Published:** 2024-03-12

**Authors:** Muriel Schraad, Stefan Runkel, Walter Hitzler, Maria Protopapa, Stefan Bittner, Timo Uphaus, Frauke Zipp

**Affiliations:** 1 Department of Neurology, Focus Program Translational Neuroscience (FTN) and Immunotherapy (FZI), Rhine Main Neuroscience Network (rmn2), University Medical Centre of the Johannes Gutenberg University Mainz, Langenbeckstr. 1, 55131 Mainz, Germany; muriel.schraad@unimedizin-mainz.de (M.S.); maria.protopapa@unimedizin-mainz.de (M.P.);; 2 Department of Transfusion Medicine, University Medical Centre of the Johannes Gutenberg University Mainz, Langenbeckstr. 1, 55131 Mainz, Germany; stefan.runkel@unimedizin-mainz.de (S.R.); hitzler@uni-mainz.de (W.H.)

**Keywords:** SARS-CoV-2, vaccination, multiple sclerosis, efficiency, booster vaccination, sphingosine-1-phopshate receptor modulator, anti-CD20

## Abstract

Immunomodulatory and immunosuppressive therapy is needed in people with a chronic neuroinflammatory disease of the central nervous system such as multiple sclerosis (MS). Therefore, MS requires monitoring for and preventing against infectious diseases like SARS-CoV-2. Vaccination and anti-viral treatments are, in particular, recommended for elderly people and people at risk of a severe course of infection and of MS. Here, we asked whether repetitive infection or vaccination influenced responses upon receiving high efficacy treatments, namely sphingosine-1-phosphate receptor modulator (S1P) or anti-CD20 B cell antibody (anti-CD20) treatments. We performed a prospective real-world study of people with MS (pwMS) under S1P or anti-CD20 with repetitive exposure to the SARS-CoV-2 virus or vaccine. The measurement of anti-SARS-CoV-2 antibody titres was performed by two independent immunoassays after initial immunisation and after booster vaccination or infection. Other laboratory and clinical parameters were included in the analysis of influencing factors. As secondary outcomes, lymphocyte and immunoglobulin levels were observed longitudinally under intravenous and subcutaneous anti-CD20 treatment. In a long-term real-world cohort of 201 pwMS, we found that despite lymphopenia upon S1P drugs, the SARS-CoV-2 immunisation response increased both in selective and non-selective S1P (100% and 88% seroconversion, respectively), whereas those under anti-CD20 therapies merely exhibited a slight long-term increase in antibody titres (52% seroconversion). The latter was independent of immunoglobulin or total lymphocyte levels, which mostly remained stable. If the individual was immunised prior to therapy initiation, their levels of SARS-CoV-2 antibodies remained high under treatment. PwMS under non-selective S1P benefit from repetitive vaccination. The risk of an insufficient vaccination response mirrored by lower SARS-CoV-2 antibodies remains in pwMS receiving anti-CD20 treatment, even after repetitive exposure to the vaccine or virus. Due to the compromised vaccination response in CD20-depleting drugs, prompt antiviral treatment might be necessary.

## 1. Introduction

The COVID-19 pandemic influenced emergency medicine services and the utilization of healthcare resources, and was associated with increased complications during hospital stays, mainly due to visitation restrictions, predominantly in vulnerable patient cohorts [1,2,3]. In addition, during the COVID-19 pandemic, people with multiple sclerosis (pwMS) exhibited a greater risk of severe disease courses associated with risk factors such as progressed disability and immune suppression [4,5]. Not only the infection itself, but also the possibly triggered disease activity resulting in clinical relapses and the progression of disability put pwMS at increased risk [6,7,8,9]. Thus, the prevention of SARS-CoV-2 infection remains critically important. Currently, authorities recommend an initial immunisation with at least two vaccination doses and one additional exposure to the virus or a third vaccination dose for everyone. Individuals with an increased risk of a more severe disease course are recommended to receive yearly booster vaccinations with virus-adapted vaccines, to be primarily performed in autumn. For those with a limited immune response, further booster doses can be indicated earlier. However, there is still a debate as to whether MS in general as a chronic illness or the intake of an immunomodulatory therapy, no matter which one, is associated with such a high risk of a severe disease course of COVID-19. Or, instead, is a more detailed description of high risk subpopulation in regards to pwMS needed?

**Figure 1:**
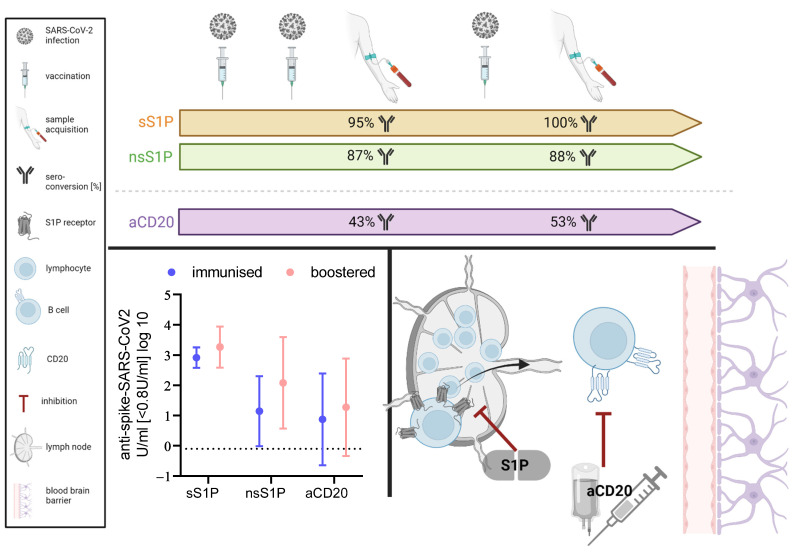
Schematic Overview. Study design with sample acquisition performed after initial immunisation and after booster vaccination or virus exposure. Within the timelines the proportion of seroconverted pwMS under sS1P (orange), nsS1P (green), and anti-CD20 (aCD20; lilac) are displayed. Anti-spike antibody levels (log10) under sS1P, nsS1P, and aCD20 after initial immunisation (blue) and after booster vaccination or infection (pink). Mean (dot) with standard deviation (bars). Lower limit of seroconversion (0.8 U/mL) displayed as its logarithm base (log10) through dotted line. Schematic display of modes of action of S1P and aCD20. S1P functionally antagonises S1P receptors and thus prevents egress of lymphocytes from lymph nodes. Anti-CD20 results in depletion of CD20-positive B cells. Reduced counts of lymphocytes and B cells in blood result in decreased autoinflammation in the central nervous system. Created with Biorender.

It is reported that the vaccination response against SARS-CoV-2 is reduced in pwMS receiving non-selective sphingosine-1-phosphate receptor modulator (ns-S1P) and anti-CD20 B cell antibody (anti-CD20) treatment [10]. The sphingosine-1-phosphate receptor modulator acts through functionally antagonising the S1P receptor on lymphocytes and thus preventing their egress from lymph nodes, resulting in substantially reduced counts of lymphocytes. They can be further subdivided into selective S1P modulators, acting on S1P receptors 1 and 5, and non-selective S1P modulators, which act on S1P receptors 1, 3, 4, and 5. CD20 antibodies, intravenously or subcutaneously applied, induce the cell death of B cells by binding to the surface marker CD20, which is present on almost all B cell subtypes except pre-B cells and plasma cells. Both result in a decrease in auto-inflammatory action within the central nervous system, decreasing the likelihood of MS relapses or disability progression from continuous inflammation. A schematic display of the modes of action of both drug groups can be found in Figure 1. Anti-CD20 treatments, especially in the long-term, result in a deficiency in immunoglobulin, whilst S1P’s primary treatment effect is lymphopenia. This is of great importance since long-term immunomodulatory therapy, like that needed in MS, is commonly associated with infections such as SARS-CoV-2 [11,12]. 

Previous studies have confirmed a greater risk of a breakthrough infection with lower anti-SARS-CoV-2-specific antibody levels and under certain disease-modifying therapies (DMTs) [13,14,15,16]. Especially under anti-CD20, the COVID-19 disease course was shown to remain more severe even after initial immunisation [15,17]. In turn, antiviral treatment with nirmatrelvir/ritonavir (Paxlovid^®^) has shown some success in preventing severe disease courses of SARS-CoV-2 infection. This includes a few cases that reported the successful use of nirmatrelvir/ritonavir in people with oncological or rheumatological illnesses treated with anti-CD20 [18,19,20]. One reported case with multiple sclerosis under ocrelizumab was successfully treated with a combination of remdesivir and nirmatrelvir/ritonavir [21]. However, access might be limited to this treatment, and its effectiveness is particularly proven within the first five days of infection, calling for increased awareness and early detection of patients at risk [22]. Furthermore, co-morbidities and other factors predicting a severe disease course as well as co-medication, which might be influenced through the cytochrome CYP3A4-inhibition potential of ritonavir, should be taken into account. 

Vaccines against SARS-CoV-2 encode SARS-CoV-2 spike protein either through mRNA encapsulated in lipid nanoparticles or adenovirus vectors [23,24]. They are then engulfed by dendritic cells through endocytosis. Recognition of mRNA or double-stranded DNA triggers the intracellular translation of spike proteins presented as antigens, sparking a SARS-CoV-2 spike-specific T cell response and B cell development into antibody-releasing plasma cells [23,24]. 

This work aims to assess, in a real-world scenario, the immune response to SARS-CoV-2 and to evaluate the benefit of booster vaccinations, in particular in pwMS receiving S1P modulators or anti-CD20 therapy, which are debated to cause a particularly high risk. Secondarily, it aims to portray immunoglobulin levels between intravenous and subcutaneous anti-CD20 in a real-world scenario.

## 2. Materials and Methods

### 2.1. Sample Acquisition

PwMS treated with S1P (selective (s-S1P) and non-selective) or anti-CD20 (ocrelizumab, rituximab) were recruited at the outpatient clinic of the Department of Neurology at the University Medical Center Mainz (Mainz, Germany) from October 2021 to July 2023 as part of the standard laboratory examination. Vaccination was performed off-site and vaccination response was analysed by electrochemiluminescence immunoassay. Clinical characteristics including sex, age, disease phenotype, DMTs, and duration of current treatment, as well as immune status, including lymphocyte composition, were obtained from standardized routine examinations. All pwMS included must have been vaccinated against SARS-CoV-2 with one of the mRNA or vector vaccines approved in Germany. They must have been at least 18 years old and treated with one of the specified DMTs. When investigating pwMS immunised prior to initiation of S1P or anti-CD20 treatment, only those who had received no DMTs, platform (interferon, glatiramer acetate, dimethyl fumarate, teriflunomide), or the solely selective, not general immunosuppressive, agent natalizumab were included, as these have been shown to have no influence on vaccination response [10]. For repetitive virus exposure, those pwMS who had experienced infection or received another vaccination were included. Information on vaccine type, time point of immunisation, booster, and infection were obtained from a standardized patient self-reported questionnaire. Certain samples were missing information regarding the type or timing of vaccination or infection. These samples were appropriately excluded from the specific statistical analyses that required this information but were still considered in the overall analyses. In regards to booster effect, vaccination or infection counted as exposure to the virus. Most patients had only experienced one or the other; however, some had gone through an infection and received a booster vaccination between initial acquisition and consecutive titre measurement. This study adhered to the principles of the Declaration of Helsinki and received approval from the local ethics committee (2019-14758_1). Written informed consent was obtained from all participants.

A schematic presentation of the study design is included in Figure 1, displaying initial serum acquisition after a complete immunisation including two vaccines and consecutive acquisition after another exposure to the virus.

### 2.2. Antibody Measurement

Anti-SARS-CoV-2 antibody measurement was performed as described in our previous work [10]. An electrochemiluminescence immunoassay (Elecsys Anti-SARS-CoV-2-S, Roche, Basel, Switzerland) and a chemiluminescent microparticle immunoassay (Abbott, Chicago, IL, USA) were performed according to the manufacturer’s protocol to detect SARS-CoV-2 IgG and IgM against spike (quantitative and qualitative) and nucleocapsid proteins (qualitative). Quantitative analysis with the electrochemiluminescence immunoassay (Roche) also included anti-spike IgA. Spike protein antibodies represent vaccination responses; thus, we focused on these. Tests on serum were performed within 5 h after blood collection. 

Blinding was achieved by distributing responsibility for recruiting, sample acquisition, measurement, and statistical analysis to separate people.

### 2.3. Statistical Analysis

Graph Pad Prism 8 and SPSS 27 were used for statistical analysis. Following a distribution analysis to assess the normality of the values using the Shapiro–Wilk test, subsequent statistical analyses included analysis of variance (ANOVA), *t*-tests, or the Kruskal–Wallis test as appropriate. All results were adjusted for multiple comparisons, and significance was determined at a threshold of *p*-values < 0.05. In evaluating the impact of various independent variables on vaccination titre, a multiple regression model was utilized. The dependent variable was transformed using the logarithm base 10 (log10) to ensure linearity. This analysis specifically included only pwMS vaccinated under S1P or anti-CD20 to identify any confounding effects. For comprehensive details on the statistical analysis, refer to the Supplementary Materials.

### 2.4. Role of the Funding Source

The funding source played no role in the design, data collection, analysis, interpretation, or report writing of this study, nor was it involved in the decision to submit the paper for publication.

## 3. Results

We collected samples of 201 individuals in this prospective study as part of our standardised routine investigations. Their characteristics can be found in Table 1. Out of these: 62 received S1P (32 ns-S1P, 30 s-S1P) and 139 received anti-CD20 (95 ocrelizumab, 15 rituximab, and 29 ofatumumab). The booster vaccinations were always performed with an mRNA vaccine. We collected samples of 82 pwMS after immunisation and consecutive exposure to the virus. These included 17 under ns-S1P, 13 under s-S1P, and 52 under anti-CD20.

The displayed combined spike antibody (IgG, IgM and IgA) levels were detected by an electrochemiluminescence immunoassay (Roche). Its high specificity and sensitivity, as well as the close correlation of both used immunoassays, has been shown before [10,25].

**Figure 2:**
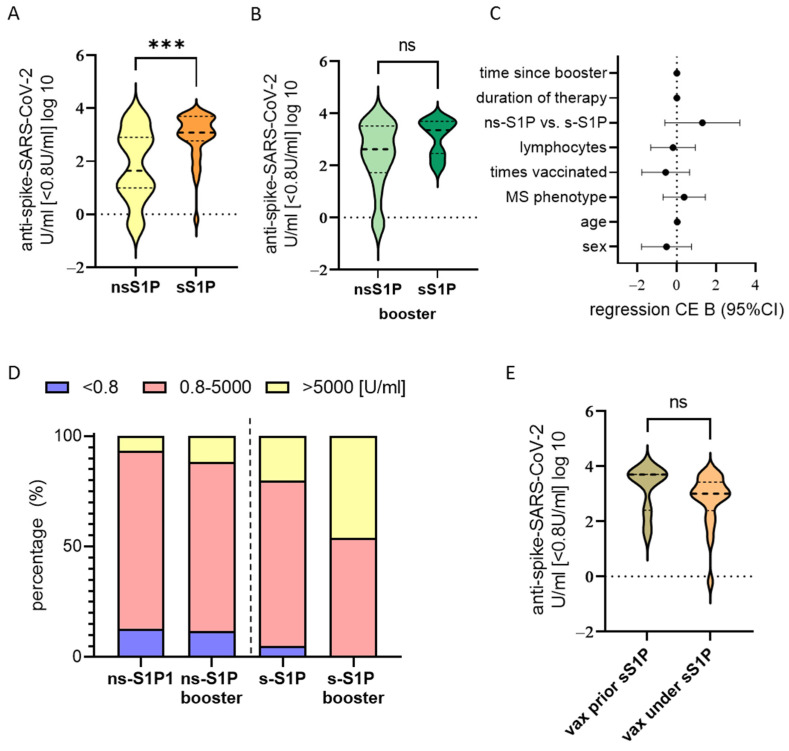
Difference in anti-spike SARS-CoV-2 vaccination titre between pwMS treated with ns-S1P and s-S1P decreases with repetitive vaccination. (**A**) Antibody levels (log10) after initial immunisation are significantly lower in pwMS under ns-S1P than under s-S1P. (**B**) With a booster vaccination or infection titre reaches similar levels between S1P subgroups. (**C**) With booster vaccination no significant influences on titre were determined in a multiple regression model. Regression coefficient B (dot) with 95% confidence interval (95%CI, whisker). (**D**) Booster increases proportion of high-responder and decreases proportion of non-responder in both ns-S1P and s-S1P treated without reaching significance (ns-S1P n = 17, s-S1P n = 13). (**E**) Antibody levels (log10) are similar between pwMS that were vaccinated (vax) under s-S1P (n = 20) and those that were vaccinated (vax) prior to (n = 7) treatment. ns = not significant, *** *p* < 0.001.

While initial immunisation numbers against SARS-CoV-2 antibodies in the patient group (expanded cohort n = 62 compared to our previous report n = 33 [10]) receiving ns-S1P were significantly lower than in those under s-S1P (*t*-test; difference of mean: 1.775; 95%CI: 0.3536, 1.701; *p* = 0.0035) (Figure 2A); the titre levels under s-S1P and ns-S1P did not differ after repetitive vaccination or exposure to the virus (Figure 2B). 

Furthermore, a multiple linear regression including pwMS treated with S1P after initial immunisation was performed to detect influences on SARS-CoV-2 spike antibodies. The number of times vaccinated (regression coefficient B: 0.674; 95%CI: 0.263, 1.085; *p* = 0.002), previous SARS-CoV-2 infection (B: 1.610, 95%CI: 0.434, 2.785; *p* = 0.009), and the lymphocyte counts (B: 1.575, 95%CI: 0.306, 2.844; *p* = 0.0016) had a beneficial impact, whilst treatment with ns-S1P negatively influenced the vaccination response (B= −1.163, 95%CI: −1.767, −0.558) after initial immunisation (Supplementary Figure S1 and Table S1). 

After a booster, vaccination, or infection, no significant influencing factors on antibody levels could be identified in those vaccinated under S1P (Figure 2C and Supplementary Table S2).

Categorising titres into non-responder (<0.8 U/mL), responder (0.8–5000 U/mL), and high-responder (>5000 U/mL) upon ≥1 booster vaccinations or infections after initial immunisation, the proportion of non-responders in ns-S1P dropped from 12.9% to 11.8%, with an increase in high-responders from 6.5% to 11.8%. The proportion of non-responders in those under s-S1P dropped from 5% to 0% with an increase in high responders from 20 to 46% (non-significant in Chi^2^, ns-S1P n = 17, s-S1P n = 13) (Figure 2D). This accounts for an increase from 95% to 100% seroconverted pwMS under sS1P and from 87.1% to 88.2% under ns-S1P (Figure 1).

Those pwMS who were initially immunised prior to the start of treatment with s-S1P displayed similar levels of antibodies to those that were immunised under treatment (Figure 2E).

**Figure 3:**
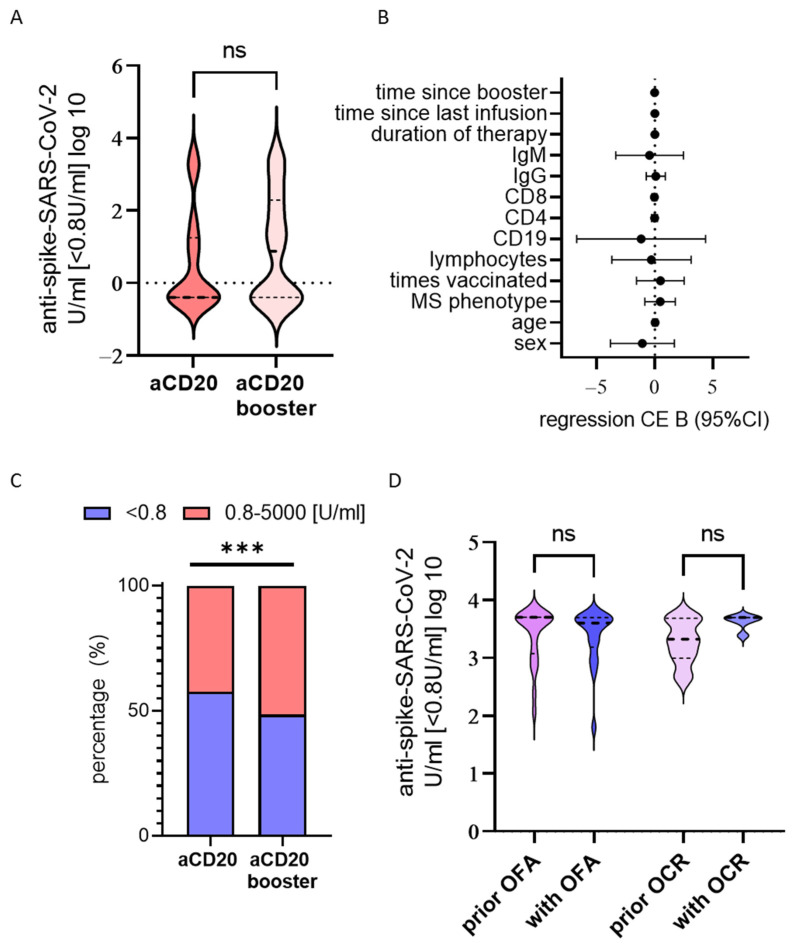
Booster response and longitudinal display of antibody levels prior and with anti- CD20 treatment. (**A**) Anti-spike SARS-CV-2 (log10) vaccination response under anti-CD20 (aCD20) after initial immunisation and after repetitive exposure to the virus (booster) shows a tendency to rise, without reaching significance. (**B**) With booster vaccination no significant influences on titre were determined in a multiple regression model. Regression coefficient B (dot) with 95% confidence interval (95%CI, whisker). (**C**) Distribution of titre divided into non-responder (<0.8 U/mL, blue), responder (0.8–5000 U/mL, pink), and high responder (>5000 U/mL, yellow) after initial immunisation and with booster. Responder increase with booster (Chi2 *** *p* < 0.001, n = 52). (**D**) Longitudinal comparison of seral titre levels (log10) acquired prior to and after initiation of therapy with ofatumumab (OFA, n = 17) or ocrelizumab (OCR, n = 6) in pwMS immunized prior to therapy initiation. ns = not significant, *** *p* < 0.001.

When initial vaccination had been performed under anti-CD20 treatment, repetitive exposure to the virus did not heighten the antibody titres significantly, but showed a tendency of rising (Figure 3A). In a multiple linear regression model, no factor significantly influencing antibody titres could be identified (Figure 3B and Supplementary Table S3). Further confirming this tendency, the proportion of non-responders dropped between the initial titre and concurrent titres after repetitive booster through vaccination or infection with SARS-CoV-2 (from 57.6% to 48.5%), with an increase in responders from 42.4% to 51.5% (Chi^2^: *p* < 0.001; n = 33) (Figure 1 and Figure 3C). 

Whilst anti-spike antibodies develop after infection and vaccination, anti-nucleocapsid antibodies solely develop after infection, as vaccines solely encode the SARS-CoV-2 spike protein. When experiencing infection, only 1 out of 16 pwMS under anti-CD20 developed quantitatively measurable IgG anti-nucleocapsid, 4 out of 16 developed IgG/IgM/IgA anti-nucleocapsid, and, at a later time point, 2 out of 14 developed IgG anti-nucleocapsid while 1 out of 14 developed IgG/IgM/IgA anti-nucleocapsid.

PwMS with initial immunisation prior to the initiation of ocrelizumab treatment display a high titre when measured after treatment start (anti-spike-SARS-CoV-2 U/mL [<0.8U/mL] log 10 mean: 2.935). In this case, the antibody levels remain sufficiently high and do not drop significantly over time (mean difference between initial titre and concurrent titre: 0.4764; 95%CI: −0.2399, 1.193; *p* = 0.1833, *t*-test). 

Furthermore, SARS-CoV-2 anti-spike antibody levels prior to the initiation of treatment with ocrelizumab or ofatumumab do not change significantly with treatment start (mean difference ocrelizumab: 0.3484; 95%CI: −0.02712, 0.7239; *p* = 0.0667; mean difference ofatumumab: −0.01695; 95%CI: −0.3375, 0.3036; *p* = 0.9153; *t*-test; Figure 3D). 

As secondary outcomes, we monitored immunoglobulin and lymphocyte levels longitudinally under anti-CD20 treatment. Within our cohort, 2.7% of patients treated with intravenous anti-CD20 developed hypogammaglobulinaemia (3/109; time point 1: 1/44 = 2.2%; time point 2: 1/39 = 2.5%; time point 3: 1/36 = 2.7%) in terms of immunoglobulin (Ig) G below the lower limit (IgG: mean 1: 10 g/L; mean 2: 9.77 g/L mean 3: 9.31; mean 4: 9.24 g/L) and in 18% of patients (20/109; time point 1: 8/44 = 18.2%; time point 2: IgM 10/39 = 22.7%; time point 3: IgM 2/36 = 5%) in terms of IgM (IgM: mean 1: 0.78 g/L; mean 2: 0.76 g/L; mean 3: 0.70 g/L, mean 4: 0.87 g/L). Out of those treated with ofatumumab, none developed hypogammaglobulinaemia. A comparison to the immunoglobulin levels in the respective approval studies ASCLEPIOS I and II for ofatumumab (reported IgG deficiency in 1.3%, IgM deficiency in 14.3%) and ORATORIO for ocrelizumab (reported IgG deficiency in 5%, IgM deficiency in 29%) [26,27] is displayed in Supplementary Figure S2. 

Lymphocyte levels also remained steady under intravenous anti-CD20, with a small proportion of patients with lymphopenia grade I [0.8–1.0/nL] (7.6%; 30/394; mean 1: 1.309/nL; mean 2: 1.349/nL; mean 3: 1.309/nL; mean 4: 1.381/nL) and even fewer with grade II [0.6–0.8/nL] (1.5%; 6/394). Under ofatumumab 23% (6/26; mean 1: 1.59/nL, mean 2: 1.4/nL) experiencing lymphopenia, including 11% (3/26) with grade II lymphopenia but none higher than that. A display of the proportion of lymphopenia in our cohort and comparison to the reported proportion of lymphopenia in the approval study for ocrelizumab ORATORIO is displayed in Supplementary Figure S3. 

Longitudinally, no major drop in immunoglobulin levels or lymphocytes could be established, but a tendency of decrease within the observed timeframe could be. The longitudinal courses of lymphocytes and immunoglobulins are displayed in Supplementary Figure S4.

## 4. Discussion

The introduction of S1P modulators has been a success in the treatment of not only relapsing-remitting MS but also in the treatment of secondary progressive MS [28,29]. In addition to siponimod, ozanimod and ponesimod functionally antagonise S1P receptors 1 and 5, whilst the first-generation S1P fingolimod functionally antagonises S1P receptors 1, 3, 4, and 5. We previously showed that even though lymphopenia was comparable between pwMS treated with non-selective and those treated with selective S1P, the antibody response to SARS-CoV-2 vaccination was lower under ns-S1P [10]. Treatment with ns-S1P had a negative influence on antibody levels after an initial vaccination course, whilst pwMS under s-S1P developed anti-SARS-CoV-2 spike antibody levels comparable to untreated pwMS and healthy controls [10,30].

Here, we demonstrate that repetitive vaccination does elevate vaccination titres in pwMS treated with non-selective S1P. As such, with repetitive vaccination, titres reach levels that are not significantly less than those in pwMS treated with selective S1P, resulting in titres similar to those of untreated pwMS and healthy controls, as outlined above. Therefore, we strongly argue against previous statements that recommend withholding vaccination in ns-S1P as, especially with repetitive vaccination, sufficient antibody levels are secured [31]. Whereas previous work described an improved antibody response with increasing lymphocytes after the cessation of ns-S1P intake [32], we find a sufficient development of anti-SARS-CoV-2 antibody titres after repetitive exposure to the virus despite continued treatment. Our finding is supported by smaller observations mentioning an increasing titre under the ns-S1P fingolimod [33,34,35]. This is of importance as the previously described T cell response under anti-CD20 has been shown to remain sufficient after a vaccination and booster, whilst no SARS-CoV-2-specific T cell response could be identified in those under ns-S1P, even when therapy had been stopped for a couple of weeks [32,36,37]. In particular, the SARS-CoV-2-specific CD8 T cell response, which is associated with a better vaccination success and reported to be especially high in pwMS under anti-CD20, was found to be diminished under S1P treatment [10,38,39,40]. A strengthened specific B cell response mirrored by SARS-CoV-2 specific spike antibodies might further diminish the risk of infection in pwMS under ns-S1P. 

In our study, repetitive vaccination or infection with SARS-CoV-2 upon anti-CD20 treatment resulted in only a slight increase in the anti-spike protein response. This is controversially discussed among a few reports, with some reporting improved and some describing sustained low antibody levels in pwMS under anti-CD20 therapy [34,35,41,42,43,44]. The minor increase in the number of pwMS who seroconvert under anti-CD20, and the greater proportion of those who do the same under ns-S1P, mirrors the vaccination response. Previous work is contradictory as to whether this correlates with a mitigated or sustained risk of infection [16,17]. Hospitalisation was not reported to be different between treatment groups after immunisation, and most importantly, none of the patients in our cohort had to undergo hospital care [17]. Nevertheless, infection can trigger disease activity in pwMS and, as such, mild or moderate cases of SARS-CoV-2 infection are also of importance [9]. Larger cohorts longitudinally correlating the risk of relapse and disease progression with SARS-CoV-2 infection are needed. In any case, our data outline that immunisation prior to the initiation of anti-CD20 results in the long-term perseverance of a sufficient immune response. This is in line with previous reports on sustained antibody levels under anti-CD20 therapy [45,46]. Those pwMS who were initially immunised prior to the initiation of treatment with s-S1P displayed similar levels of antibodies to those that were immunised under treatment, once more confirming that treatment with s-S1P does not impact antibody development [10]. This robust immune response is in line with the lack of or only minor impact of treatment on infection rates in phase 3 trials of s-S1P [28,47,48,49]. 

There are some limitations to our study. Due to a quantitative minimum and maximum of the immunoassays used, minor numerical differences between titres under ns-S1P and s-S1P could be hidden. Of additional interest would be the vaccine-specific T cell response after the booster, which has been shown to be efficient under B cell depletion and diminished under S1P therapy after initial immunisation [36,37,39,50]. We corrected for variance in the time points of titre measurements through a multiple regression model due to the real-world observation setting in which vaccination and titre acquisition have not been coordinated in advance. Some pwMS in our cohort could not self-report all vaccination time points; furthermore, self-testing for COVID-19 decreased over time and, as such, not every infection and subclinical disease could be monitored. Electronic health records, which could have objectified self-reported information, were not available. Finally, to further investigate the vaccination response against different variants of SARS-CoV-2, longer follow-up monitoring would be of utmost importance. 

Interestingly and of further relevance to infection and vaccination in pwMS, the proportion of those with hypogammaglobulinemia in our real-world cohort was lower than in previously published studies on ocrelizumab [26]. Furthermore, only 9.1% displayed lymphopenia at any time during our observation period, which is lower than the reported 20.7% in RRMS and 26.3% in PPMS in the approval study, most of those with only mild lymphopenia [51]. However, one must take into account that our real-world cohort did include patients with variable durations of therapy whilst the comparison study was a 5-year follow-up of the approval study. Out of those treated with ofatumumab, none displayed hypogammaglobulinaemia, but 23% experienced lymphopenia [<1.0/nL], with 11% being grade 2 [0.6–0.8/nL] but none being higher than that. The approval study displayed higher levels of hypogammaglobulinaemia, especially for IgM [27]. Notably, in described studies, hypogammaglobulinaemia had been defined as 20% below the lower limit of normal for IgG and 10% below the lower limit of normal for IgM; absolute level comparison should be aimed for in future studies [27]. Nevertheless, our patients only received ofatumumab for a maximum of 335 days, in comparison to the approval study that monitored patients for up to 30 months [27,52]. Altogether, monitoring lymphocytes and immunoglobulin levels under B cell depletion therapy remains relevant as the infection risk has been shown to be associated with lymphopenia and long-term therapy that is associated with hypogammaglobulinaemia [53,54]. Long-term observation will enlighten us as to whether one or the other form of application might prevail in balancing needed immunosuppressive effects with a sustained level of immune defence. 

Overall, anti-SARS-CoV-2 antibody levels might slightly increase in pwMS under anti-CD20, whilst, as described above, those pwMS receiving ns-S1P therapy benefit immensely from booster vaccination. These findings support a recommendation to apply a personalised treatment approach for pwMS under anti-CD20 with close monitoring and early protection by antiviral therapy (nirmatrelvir/ritonavir) when infected. Yet, when prescribing nirmatrelvir/ritonavir, one needs to keep in mind that ritonavir inhibits cytochrome CYP3A4 and, thus, can interact with some medications, including siponimod, which is in part metabolised by CYP3A4. However, these interactions were described nationally and internationally as being of very low risk for adverse events or decreased efficacy. In particular, additional risk factors such as a high Expanded Disability Status Scale (EDSS) score, older age, or other comorbidities call for specific action. 

## 5. Conclusions

In summary, our study provides insights into the vaccination response of pwMS under S1P and anti-CD20 treatments. Repetitive exposure to SARS-CoV-2 through infection or vaccination results in an improved antibody response, especially in pwMS under ns-S1P and possibly in those under anti-CD20 therapy. This once more supports the application of personalized vaccination strategies, and monitoring and treatment strategies, in populations at risk for a severe infection course or potential disease worsening through infection. Furthermore, we confirm a sustained protection against infection when vaccination is performed before the initiation of anti-CD20 treatment. 

## Figures and Tables

**Table 1:** Demographic and clinical characteristics of pwMS that were included in the analysis of the initial immunisation titre and second titre. “Time lag” represents time elapsed between titre acquisition and last vaccination (days). “Booster vaccination or infection in between” indicates the number of pwMS who reported having received a booster vaccination or having been infected with SARS-CoV-2 between acquisition of the initial and subsequent titre. “Infusion to boost titre” represents time elapsed between titre acquisition and last infusion of intravenous anti-CD20 antibody (days), “infusion to boost” represents time elapsed between booster vaccination and last infusion of intravenous aCD20 vaccination (days), and “duration boost to titre” represents time elapsed between booster vaccination and titre acquisition (days). RRMS = relapsing-remitting MS, SPMS = secondary progressive MS, PPMS = primary progressive MS, CIS = clinically isolated syndrome, S1P = sphingosine-1-phosphate-modulators, ns-S1P = non-selective S1P, s-S1P = selective S1P.

**pwMS vaccinated, first titre**	**n = 201 (%)**	**mean (SD)**
**age-mean**		43.7 (12.04)
**female sex**	133 (66.17)	
**MS phenotype**		
**RRMS**	152 (75.62)	
**SPMS**	30 (14.93)	
**PPMS**	19 (9.45)	
**time lag (days)**		140.83 (115.2)
**therapy**		
**ns-S1P**	32 (15.9)	
**s-S1P**	30 (14.93)	
**ocrelizumab**	95 (47.26)	
**ofatumumab**	29 (14.43)	
**rituximab**	15 (7.46)	
**count of vaccination**		2.59 (0.72)
**vaccine type**		
**BNT162b2**	117 (58.21)	
**mRNA-1273**	7 (3.4)	
**mRNA combi**	3 (1.5)	
**vector mRNA combi**	33 (17.7)	
**duration of therapy (1) (days)**		885.33 (884.62)
**infusion to titre**		144.09 (64.83)
**pwMS vaccinated, second titre**	**n = 124 (%)**	**mean (SD)**
**therapy**		
**ns-S1P**	18 (14.5)	
**s-S1P**	18 (14.5)	
**ocrelizumab**	65 (52.42)	
**ofatumumab**	17 (13.7)	
**rituximab**	6 (4.8)	
**booster vaccination or infection in between**	82 (66.13)	
**booster vaccination**	54 (43.55)	
**BNT162b2**	28 (51.85)	
**mRNA-1273**	8 (14.82)	
**unknown booster vaccine information**	18 (33.33)	
**duration of therapy (2) (days)**		988.45 (413.605)
**infusion to boost titre**		161.05 (102.83)
**infusion to boost**		149.91 (138.073)
**duration boost to titre**		215.38 (146.97)
**time since previous titre 1**		181.21 (106.28)

## Data Availability

The data that support the findings of this study will be available upon reasonable request to the corresponding author of the study.

## Supplementary Materials

The following supporting information can be downloaded at: https://www.mdpi.com/article/10.3390/vaccines12030296/s1, Figure S1: Regression model on S1P treated pwMS.; Figure S2: Comparison of proportions of hypogammaglobulinaemia in pwMS treated with anti-CD20 of our cohort to the approval studies ORATORIO and ASCLEPIOS I & II; Figure S3: Comparison of lymphopenia vs. normal range levels of lymphocytes between Mainz cohort to approval studies; Figure S4: Immunoglobulin and lymphocyte levels over the monitoring time; Table S1: Regression model on pwMS under S1P; Table S2: Regression model on pwMS vaccinated and boostered under S1P; Table S3: Regression model on pwMS vaccinated and boostered under aCD20.
