# Supplementary material for: Long-Term Observation of SARS-CoV-2 Vaccination Response upon High Efficacy Treatment in Multiple Sclerosis—A Real-World Scenario
Source: Vaccines (Basel). 2024 Mar 12;12(3):296. doi: 10.3390/vaccines12030296 (PMC10974098; doi:10.3390/vaccines12030296)
Supplement: Supplementary file 1 [file vaccines-12-00296-s001.zip › vaccines-2880463-supplementary.pdf]

Supplementary Figures

Supplementary Figure S1

Regression model on S1P treated pwMS. After correction, titre (log10) was still significantly influenced by lymphocyte count, number of vaccinations, and treatment with nsS1P. Forest plot displaying regression coefficients (CE) B (dot) with 95% confidence interval (95% CI, whiskers) of linear regression model performed on pwMS treated with S1P and anti-spike-SARS-CoV-2 as dependent variable. *p < 0.05, **p < 0.01.

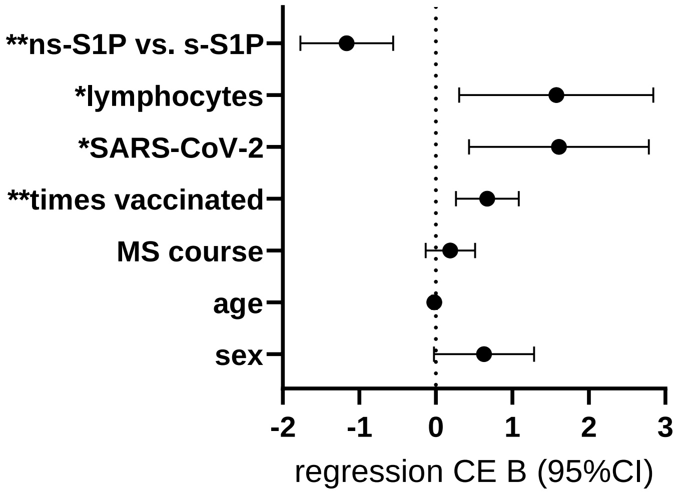

Supplementary Figure S2

Comparison of proportions of hypogammaglobulinaemia in pwMS treated with anti-CD20 of our cohort to the approval studies ORATORIO and ASCLEPIOS I & II. (A) Proportion of deficiency in IgG and (B) IgM of pwMS treated with ofatumumab (OFA) or ocrelizumab (OCR) within our cohort (Mainz) in comparison to reported levels in approval studies ASCLEPIOS I and II or ORATORIO, respectively. Deficiency was defined in our cohort and ORATORIO as all levels below lower limit of normal, whilst in ASCLEPIOS defined as 20% below or 10% below lower limit of normal for IgG and IgM, respectively.

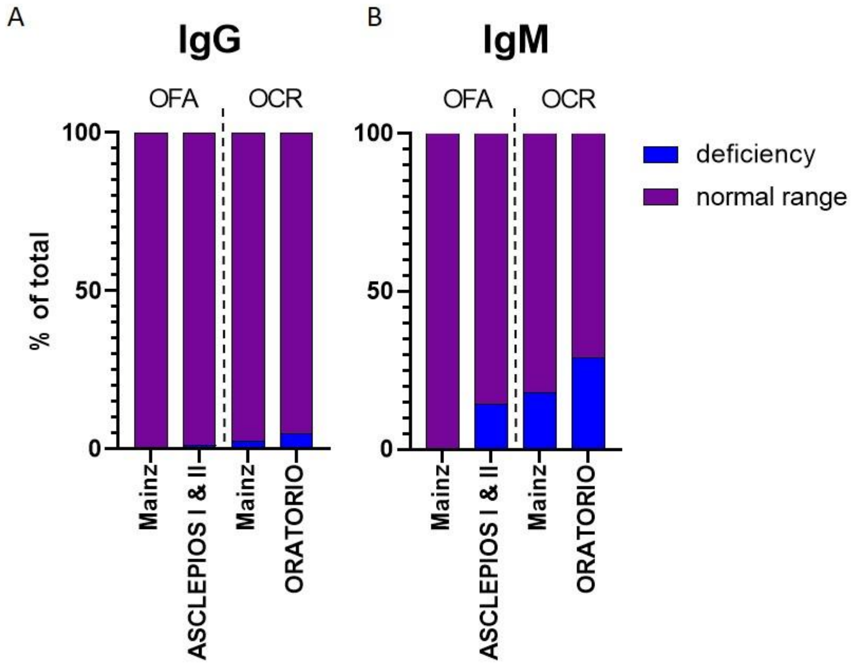

Supplementary Figure S3

Comparison of lymphopenia vs. normal range levels of lymphocytes between Mainz cohort to approval studies. Percentage of pwMS under Ofatumumab or Ocrelizumab with lymphopenia (orange; <1000/µl) and those with lymphocyte levels within the normal range of respective laboratory (green). Comparing levels between Mainz cohort to those from approval study ORATORIO for ocrelizumab. No data could be found on the percentage of lymphopenia under ofatumumab within the approval study ASCLEPIOS I & II, thus only Mainz cohort is displayed.

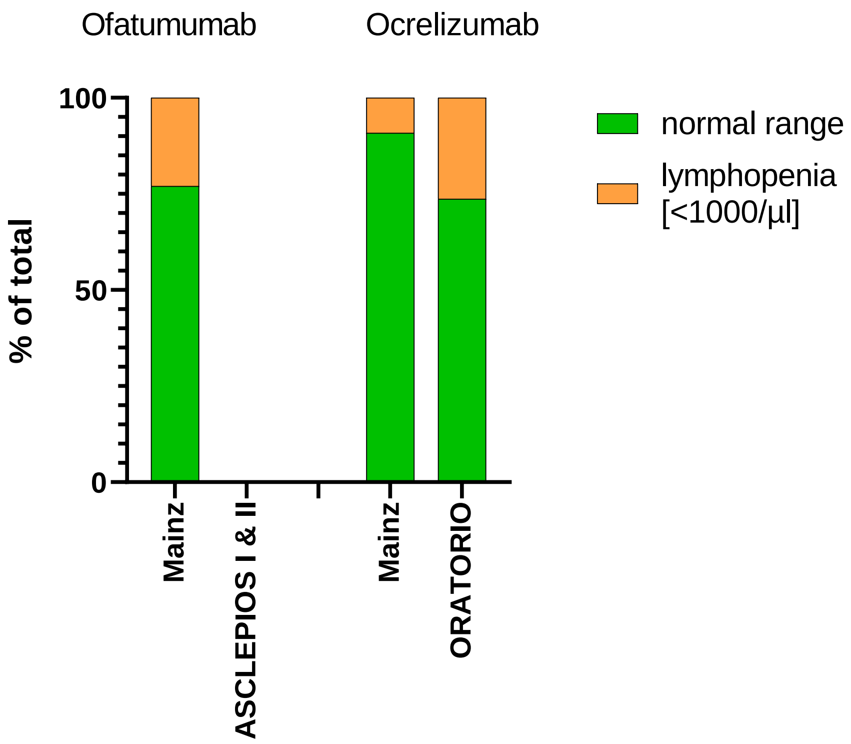

Supplementary Figure S4

Immunoglobulin and lymphocyte levels over the monitoring time. Dots identify individual pwMS related to days since the first measurement. Red dotted lines define lower and upper limit of normal or grade I, II or III lymphopenia levels.

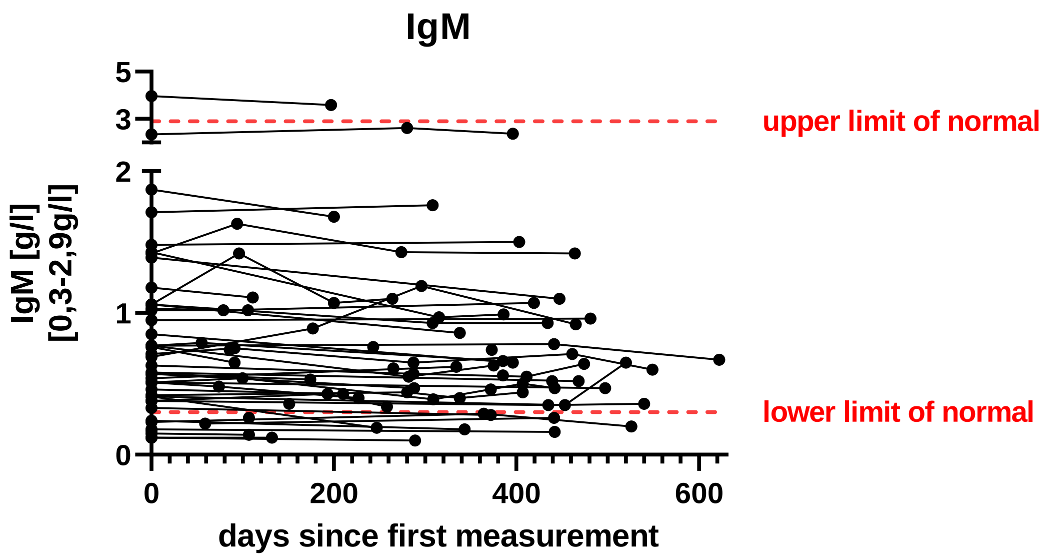

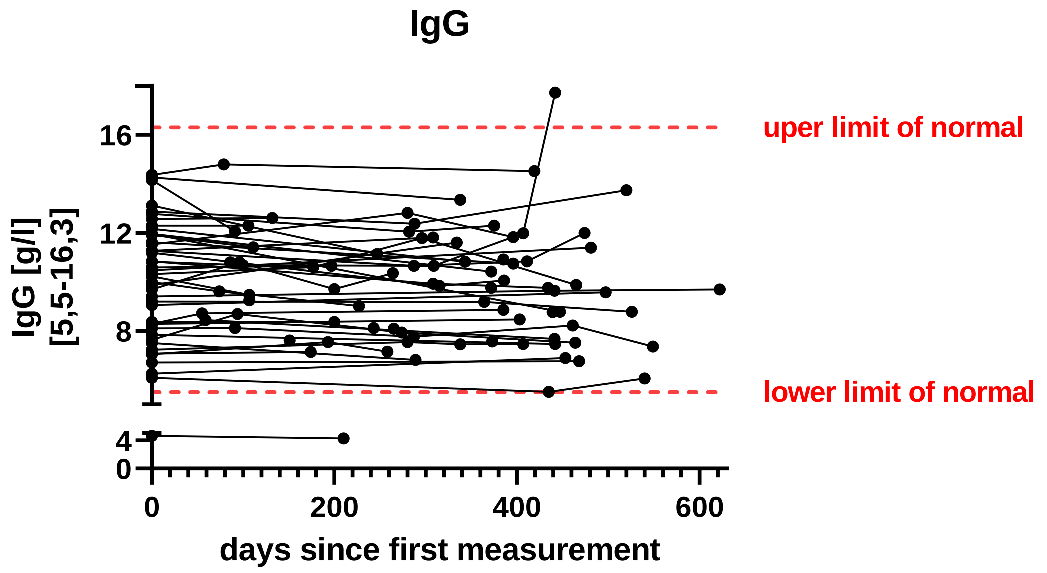

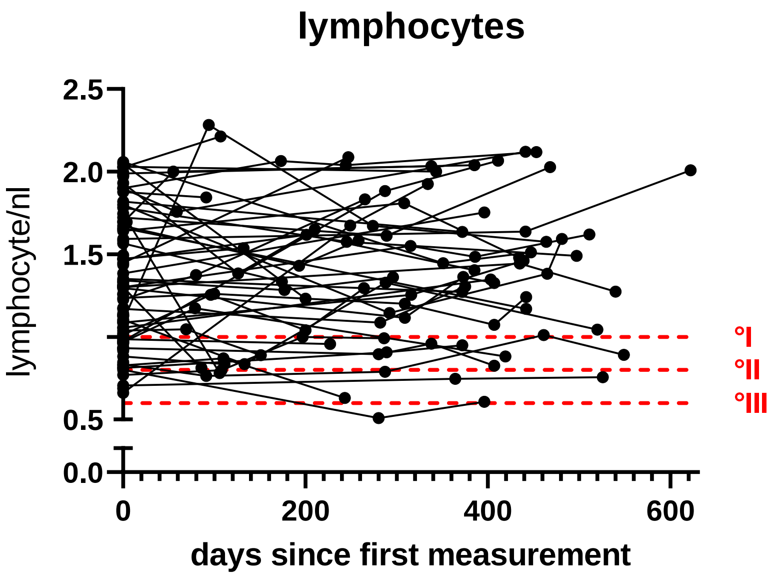

Supplementary Tables
Supplementary Table S1

Regression model on pwMS under S1P. Linear regression model of co-variables influencing anti-spike-SARS-CoV-2 (log10) displayed in **Supplementary Figure 1** performed on pwMS vaccinated under S1P. Antibody level was significantly influenced by lymphocyte count, number of vaccinations and treatment with ns-S1P in reference to those treated with s-S1P. Regression coefficients B with 95% confidence interval (upper/lower bound) and level of significance. Treatment with ns-S1P was referenced (ref.) to treatment with s-S1P. P values <0.05 were considered significant, and are indicated by bold text.

**independent variables**	**regression coefficient B**	**level of significance (p)**	**lower bound 95%CI**	**upper bound 95%CI**
sex	0,437	0,286	-0,384	1,259
age	-0,029	0,094	-0,063	0,005
disease course	0,023	0,866	-0,252	0,298
times vaccinated	0,615	0,015	0,125	1,105
s-S1P	ref.			
ns-S1P	-1,344	0,000	-2,023	-0,665
lymphocytes	1,547	0,026	0,201	2,894

Supplementary Table S2

Regression model on pwMS vaccinated and boostered under S1P. Linear regression model of co-variables influencing anti-spike-SARS-CoV-2 (log10) displayed in **Figure 1C** performed on pwMS under S1P that received a booster vaccination. No significant influences on antibody levels could be identified. Regression coefficients B with 95% confidence interval (upper/lower bound) and level of significance. Treatment with ns-S1P was referenced (ref.) to treatment with s-S1P. P values <0.05 were considered significant, and are indicated by bold text.

**independent variables**	**regression coefficient B**	**level of significance (p)**	**lower bound 95%CI**	**upper bound 95%CI**
sex	-0,525	0,404	-1,794	0,744
age	0,013	0,608	-0,039	0,066
disease course	0,366	0,490	-0,705	1,438
times vaccinated	-0,568	0,345	-1,779	0,643
S-S1P	ref.			
ns-S1P	1,292	0,174	-0,605	3,188
lymphocytes	-0,194	0,727	-1,323	0,935
time since booster	-0,002	0,392	-0,008	0,003
duration of therapy	-0,001	0,196	-0,001	0,000

Supplementary Table S3

Regression model on pwMS vaccinated and boostered under aCD20. Linear regression model of co-variables influencing anti-spike-SARS-CoV-2 (log10) performed on pwMS under aCD20 that received a booster vaccination. No significant influences on antibody levels could be identified. Regression coefficients B with 95% confidence interval (upper/lower bound) and level of significance. P values <0.05 were considered significant, and are indicated by bold text.

independent variables	regression coefficient B	level of significance (p)	lower bound 95%CI	upper bound 95%CI
sex	-1,063	0,366	-3,814	1,688
age	0,022	0,792	-0,177	0,220
course	0,460	0,411	-0,859	1,778
times vaccinated	0,486	0,569	-1,566	2,538
lymphocytes	-0,283	0,839	-3,686	3,121
CD19	-1,169	0,611	-6,706	4,368
CD4	-0,020	0,842	-0,269	0,228
CD8	-0,026	0,759	-0,228	0,177
IgG	0,087	0,795	-0,729	0,903
IgM	-0,442	0,712	-3,349	2,465
time since booster	-0,006	0,200	-0,017	0,004
time since last infusion	0,005	0,326	-0,007	0,016
duration of therapy	0,001	0,699	-0,004	0,005
